# Pyloric, pseudopyloric, and spasmolytic polypeptide-expressing metaplasias in autoimmune gastritis: a case series of 22 Japanese patients

**DOI:** 10.1007/s00428-021-03033-5

**Published:** 2021-01-30

**Authors:** Yasuhiro Wada, Shigemi Nakajima, Ryoji Kushima, Shizuki Takemura, Naoko Mori, Hiroshi Hasegawa, Takahisa Nakayama, Ken-ichi Mukaisho, Akiko Yoshida, Shinji Umano, Kazuo Yamamoto, Hiroyuki Sugihara, Kazunari Murakami

**Affiliations:** 1 Department of Pathology, Shiga University of Medical Science, Seta-tsukinowa-cho, Otsu, Shiga 520-2192 Japan; 2 Department of Gastroenterology, Japan Community Healthcare Organization (JCHO) Shiga Hospital, Consortium for Community Medicine, Shiga University of Medical Science, Otsu, Shiga Japan; 3 Department of Gastroenterology, Faculty of Medicine, Oita University, Yufu, Oita Japan; 4 Division of Diagnostic Pathology, Kusatsu General Hospital, Kusatsu, Shiga Japan; 5 Department of Pathology, Japan Community Healthcare Organization (JCHO) Shiga Hospital, Consortium for Community Medicine, Shiga University of Medical Science, Otsu, Shiga Japan

**Keywords:** Autoimmune gastritis, Pseudopyloric metaplasia, Spasmolytic polypeptide-expressing metaplasia, Gastrin

## Abstract

There are two types of pyloric gland-like metaplasia in the corpus of stomach: pyloric and pseudopyloric metaplasias. They show the same morphology as the original pyloric glands in H&E staining. Pseudopyloric metaplasia is positive for pepsinogen (PG) I immunohistochemically, whereas pyloric metaplasia is negative. Recently, spasmolytic polypeptide-expressing metaplasia (SPEM) is proposed for pyloric gland-like metaplasia mainly in animal experiments. SPEM expresses trefoil factor family 2 (TFF2) and is often considered synonymous with pseudopyloric metaplasia. We reviewed consecutive 22 Japanese patients with autoimmune gastritis (AIG) to investigate TFF2 expression in pyloric and pseudopyloric metaplasias by counting all pyloric gland-like glands in biopsy specimens taken from greater curvature of the middle corpus according to the Updated Sydney System. Pyloric metaplasia was seen in all the 22 cases, and pseudopyloric metaplasia was found in 15 cases. Of 1567 pyloric gland-like glands in all the cases, 1381 (88.1%) glands were pyloric metaplasia glands, and the remaining 186 (11.9%) glands were pseudopyloric metaplasia glands. TFF2 expression was observed in pyloric or pseudopyloric metaplasia glands in 20 cases. TFF2 expression was recognized in 409 of 1381 (26.9%) pyloric metaplasia glands and 27 of 186 (14.5%) pseudopyloric metaplasia glands (*P*<0.01, chi-square test). In conclusion, SPEM was not always the same as pseudopyloric metaplasia in human AIG, and the majority of metaplasia in AIG was not pseudopyloric but pyloric metaplasia.

**Supplementary Information:**

The online version contains supplementary material available at 10.1007/s00428-021-03033-5.

## Introduction

Metaplasia is a phenomenon in which one adult cell type is replaced by another adult cell type [1]. In stomach, various types of metaplasias have been described. Intestinal metaplasia is one of the representative metaplasias in the stomach which consists of large or small intestinal mucosa located in the places where gastric mucosa should have existed [1]. Pyloric/pseudopyloric metaplasia is another representative metaplasia which consists of pyloric gland mucosa located in the places where fundic gland mucosa should have existed [2–4]. Pancreatic acinar cell metaplasia is a cluster composed of pancreatic acinar cells in stomach [5–7].

Pyloric metaplasia is stained same as original pyloric gland mucosa with hematoxylin and eosin (H&E) and with immunohistochemical staining for MUC6. Pseudopyloric metaplasia is also stained the same as original pyloric gland mucosa with H&E but positive for pepsinogen (PG) I immunohistochemically, whereas pyloric metaplasia is negative [3]. Unfortunately, in many papers, pyloric and pseudopyloric metaplasias have not been described separately and are often treated as collectively “pyloric or pseudopyloric metaplasia” [4].

Recently, trefoil factor family 2 (TFF2) expression in the stomach of animals has been used to designate something like spasmolytic polypeptide-expressing metaplasia (SPEM). Wang et al. have reported that an aberrant metaplastic lineage was found in fundic mucosa of mice infected with *Helicobacter felis*, and this metaplastic lineage expressed TFF2, reflecting regenerative changes in gastric mucosa [8–10]. Several reports have indicated that SPEM is a precancerous lesion of gastric carcinoma [11–14], whereas Graham and Zou suggested that the direct experimental evidence of SPEM to gastric cancer transition was minimal, and SPEM should not be used for studies in humans [15]. In many papers, SPEM is often considered synonymous with pseudopyloric metaplasia [16–20]. However, in *Helicobacter pylori* (*H. pylori*) gastritis, pyloric and pseudopyloric metaplasias did not always express TFF2 [4].

Autoimmune gastritis (AIG) is a chronic, progressive inflammatory disease which is characterized with marked destruction of parietal cells in gastric mucosa by autoimmune mechanisms resulting in decreased gastric acid secretion followed by hypergastrinemia via negative feedback reaction [21–23]. As AIG progresses, severe atrophy of fundic glands is also accompanied with various kinds of metaplasia, such as intestinal metaplasia, pyloric/pseudopyloric metaplasia, and pancreatic acinar cell metaplasia [24–30]. Therefore, AIG is one of the models to examine TFF2 expression in pyloric/pseudopyloric metaplasia.

In this study, we examined the numbers of TFF2 expressing glands in pyloric/pseudopyloric metaplasia glands and confirmed whether SPEM can be used for pseudopyloric metaplasia in human materials.

## Materials and methods

### Subjects

We retrospectively reviewed the biopsy specimens of consecutive 22 patients (7 male and 15 female) who underwent esophagogastroduodenoscopy and who were clinically and histopathologically diagnosed as AIG in Japan Community Healthcare Organization (JCHO) Shiga Hospital, Japan, between May 2012 and January 2020. Diagnosing criteria for AIG consisted of positivity of either serum anti-parietal cell or anti-intrinsic factor antibodies, accompanied with at least one of the following four items: increased serum gastrin, severe endoscopic gastric mucosal atrophy, strongly positive (3+) serum PG test, and decreased serum vitamin B_12_, and also being satisfied with histopathological criteria as described later. Serum gastrin, PG, and vitamin B_12_ were measured with Gastrin RIA Kit II (Fujirebio), ARCHITECT Pepsinogen I and II (Abbott Japan, Tokyo, Japan), and Beckman Coulter ACCESS B_12_ (Beckman Coulter), respectively.

In order to confirm the infection status of *H. pylori*, history about the past *H. pylori* tests and therapies was collected from the medical records. *H. pylori* tests included serum *H. pylori* antibody test (HpAb) (E-plate Eiken *H. pylori* antibody II, Eiken Kagaku, Tochigi, Japan), *H. pylori* stool antigen test (HpSA) (Meridian HpSA ELISA II, Fujirebio), urea breath test (UBT) (Ubit Tablets 100 mg, Otsuka Pharmaceutical, Tokyo, Japan; POCone, Otsuka Electronics, Osaka, Japan), histopathology (H&E staining and Giemsa staining), and culture for *H. pylori*. Serum anti-*H. pylori* was tentatively judged negative when the ELISA value was below 10.0 U/mL according to the manufacturer’s instruction. HpSA was tentatively judged positive with the value 0.120 or more and negative with the values less than 0.100. UBT was tentatively judged negative with the delta-^13^CO_2_ less than 2.0‰ and positive with the value 5.0‰ or more. Patients with clear evidence of current *H. pylori* infection with pathology or culture were determined “currently infected.” Those showing no evidence of infection by all the tests performed were diagnosed “not infected.” Those who had past eradication therapy were included in “past infected.” Other patients were diagnosed either of the above three status from the combination of multiple tests and their chronological changes. Therefore, patients with current *H. pylori* infection were not included in this study.

### Histopathological examination of AIG

Biopsy specimens were collected from greater curvature of the middle corpus and antrum endoscopically by three-points biopsy or five-points biopsy. In three-points biopsy, we collected biopsy specimens from greater curvature of the middle corpus and antrum and lesser curvature of the angulus. In five-points biopsy, we collected biopsy specimens from greater curvature of the middle corpus and antrum, and lesser curvature of the middle corpus, angulus, and antrum according to the Updated Sydney System (USS). Among them, we evaluated specimens taken from greater curvature of the middle corpus and antrum in this study. USS proposes that “B2” and “A2” are the best biopsy sites to evaluate the histopathological status of fundic and antral mucosa, respectively [31]. Biopsy specimens of all patients were taken once per patient, and each specimen was put into separate vials. Biopsy specimens were immediately fixed in 10% neutral buffered formalin for 24 h and embedded in paraffin. Fixed samples were serially sliced into 3-μm-thick sections and stained with H&E. According to the previous reports, the histopathology was classified into three stages with H&E-stained sections: early, florid, and end stages [24–28]. The early stage consisted of moderate inflammatory cell infiltration in the lamina propria and decreased parietal cells. The florid stage consisted of marked atrophy of fundic glands with diffuse lymphoplasmacytic infiltration. The end stage consisted of reduced inflammatory cell infiltrates and complete parietal cell loss [32]. AIG was comprehensively diagnosed by two pathologists with more than 20 years of diagnostic experience.

Inflammation, activity, atrophy, and intestinal metaplasia of greater curvature of the middle corpus were evaluated with the USS (0, none; 1, mild; 2, moderate; 3, marked) [31]. In addition, the lymphoid follicles and lymphocyte aggregation (LF/LA) were also examined and scored (0, none; 1, small lymphocyte aggregation; 2, 1 or 2 lymphoid follicles were observed; and 3, more than 3 lymphoid follicles were observed).

### Evaluation of AIG with immunohistochemical staining

In AIG, gastrin cell hyperplasia is often recognized in the antrum [3]. On the other hand, linear and nodular types of enterochromaffin cell-like (ECL) cell hyperplasia are seen in the corpus in florid or end stages [26, 33]. Linear type is recognized as clusters of hyperplastic ECL cells lining in the glands and nodular type as clusters of ECL cells out of the glands in the lamina propria. In recent years, BCL-10 is reported to be positive in pancreatic acinar cell metaplasia [34, 35]. We counted the numbers of H^+^/K^+^-ATPase-positive cells, ECL cells, and gastrin cells per each section. Then, we measured the size of each section and we calculated the cell density per each section. Thus, the densities of parietal cells, ECL cells, and gastrin cells and the presence of pancreatic acinar cell metaplasia were examined in each immunohistochemically stained section.

For immunohistochemical examination, the following antibodies were used as primary antibodies: anti-H^+^/K^+^-ATPase α (1:10000; Medical & Biological Laboratories, Nagoya, Japan, cat. no. 024) for parietal cells, anti-chromogranin A (diluted; Roche, Basel, Switzerland, cat.760-2519) for ECL cells, anti-BCL-10 (1:100; Santa Cruz Biotechnology, Santa Cruz, CA, USA, cat. SC-5273) and anti-α-amylase (1:1000; Cell Signaling Technology, Danvers, MA, USA, cat. no. 3796) for pancreatic acinar cell metaplasia, and anti-gastrin (diluted; Dako, Glostrup, Denmark, cat. IR519) for gastrin cells. An automated immunostaining device was used for immunostaining (Ventana; Discovery XT, Roche Diagnostics K.K., Tokyo, Japan). Immunohistochemical examination was performed at Shiga University of Medical Science. Image analysis software (WinROOF2018; Mitani Corporation, Fukui, Japan) was used to measure the size of the section.

### Evaluation of pyloric gland-like metaplasias

In the corpus mucosa, we classified pyloric gland-like glands into pyloric and pseudopyloric metaplasias. Briefly, anti-MUC6 (diluted; Cell Signaling Technology, cat.760-4390) and anti-PGI (1:40; BIO RAD, Hercules, CA, USA, cat.7240-1009) antibodies were used for the immunohistopathological differentiation of these metaplasias. Pyloric and pseudopyloric metaplasias were identified as in the following: MUC6-positive/PGI-negative for pyloric metaplasia and MUC6-positive/PGI-positive for pseudopyloric metaplasia. We also examined TFF2 expression in pyloric and pseudopyloric glands by anti-TFF2 antibody (1:600; Proteintech, Rosemont, IL, USA, cat.D031-3) with immunohistochemical staining. We counted the numbers of pyloric and pseudopyloric metaplasia glands in all the sections. Then, the numbers of TFF2 expressing glands in pyloric and pseudopyloric metaplasias were counted in all the sections.

### Statistical analysis

The Mann-Whitney test was used to evaluate the densities of H^+^/K^+^-ATPase-positive parietal cells, ECL cells, and gastrin cells. The chi-square test was used to evaluate the TFF2-positive gland ratio between pyloric and pseudopyloric metaplasias. SPSS (Stats Guild Inc. Chiba, Japan) was used in each analysis, and a value of *P*<0.05 was considered significant.

## Results

### Histopathological evaluation of AIG

**Fig. 1:**
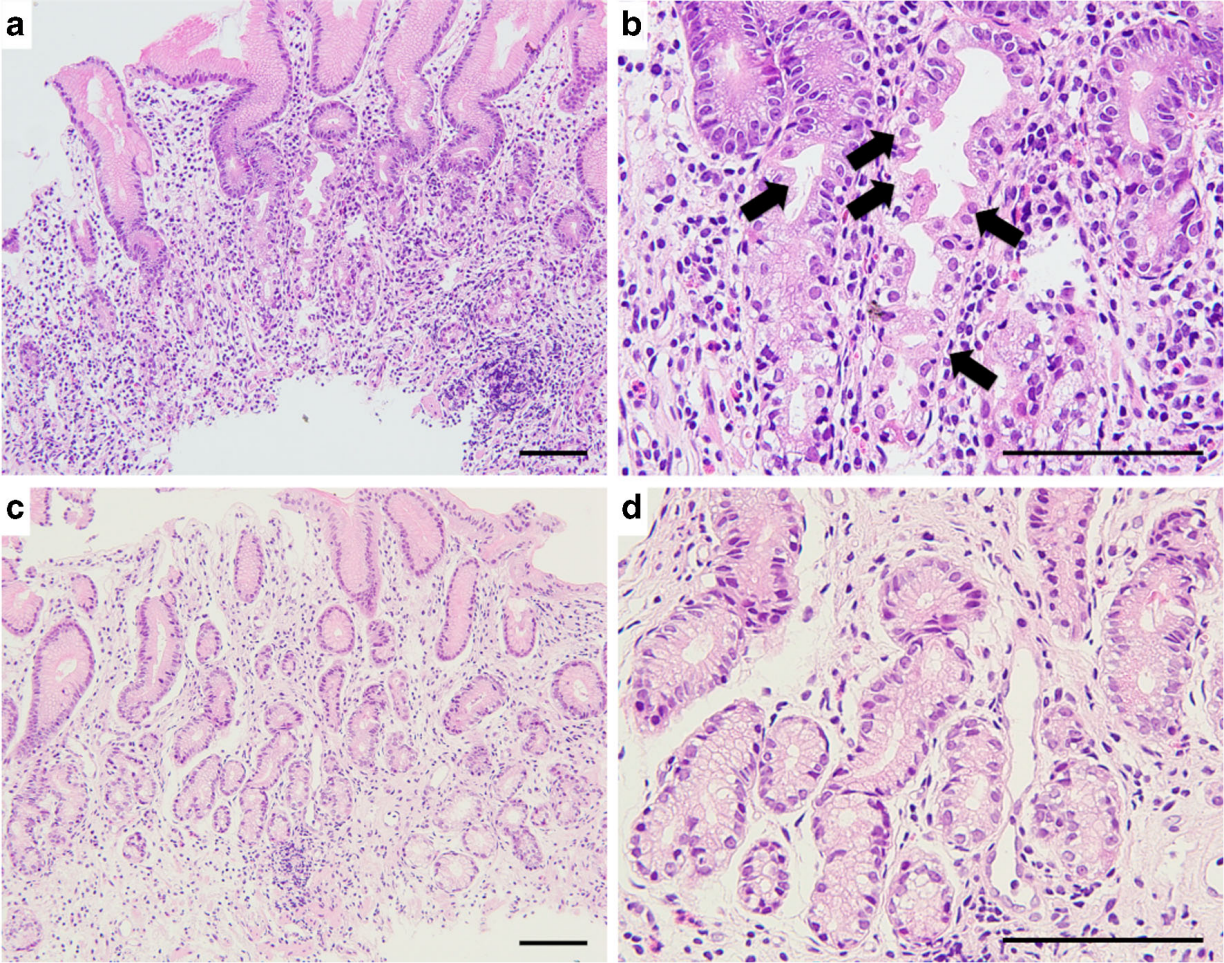
Light microscopic pictures of the typical AIG cases in florid and end stages (H&E stain, low (**a**, **c**) and high (**b**, **d**) power magnifications). In the florid stage, the parietal cells are extensively reduced and persistent inflammation is observed (**a**). Parietal cells are under destruction (**b**: arrow). In the end stage, parietal cells are completely lost and inflammatory cell infiltrates are reduced (**c**). Pyloric-like glands are seen in the bottom of the mucosa (**d**). Scale bar: 200μm

**Fig. 2:**
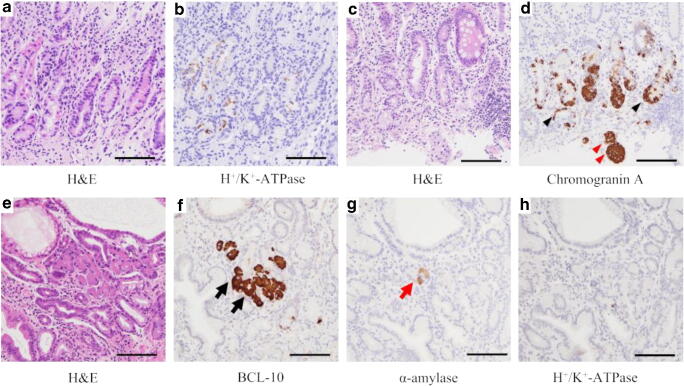
Light microscopic pictures of the typical AIG cases with H&E (**a**, **c**, **e**) and immunohistochemical stainings with anti- H^+^/K^+^-ATPase (**b**, **h**), anti-Chromogranin A (**d**), anti-BCL-10 (**f**) and anti-α-amylase (**g**). Parietal cells are significantly reduced in the oxyntic mucosa in a case of florid stage (**a**, **b**). ECL cell hyperplasia is recognized in the corpus mucosa in linear (black arrowhead) and nodular types (red arrowhead) in a case of florid stage (**c**, **d**). Pancreatic acinar cell metaplasia is found in the oxyntic mucosa of a case in end stage which showed BCL-10 positive (**f** black arrow), α-amylase vague (**g** red arrow), and H^+^/K^+^-ATPase negative (**h**). Scale bar 200μm

**Fig. 3:**
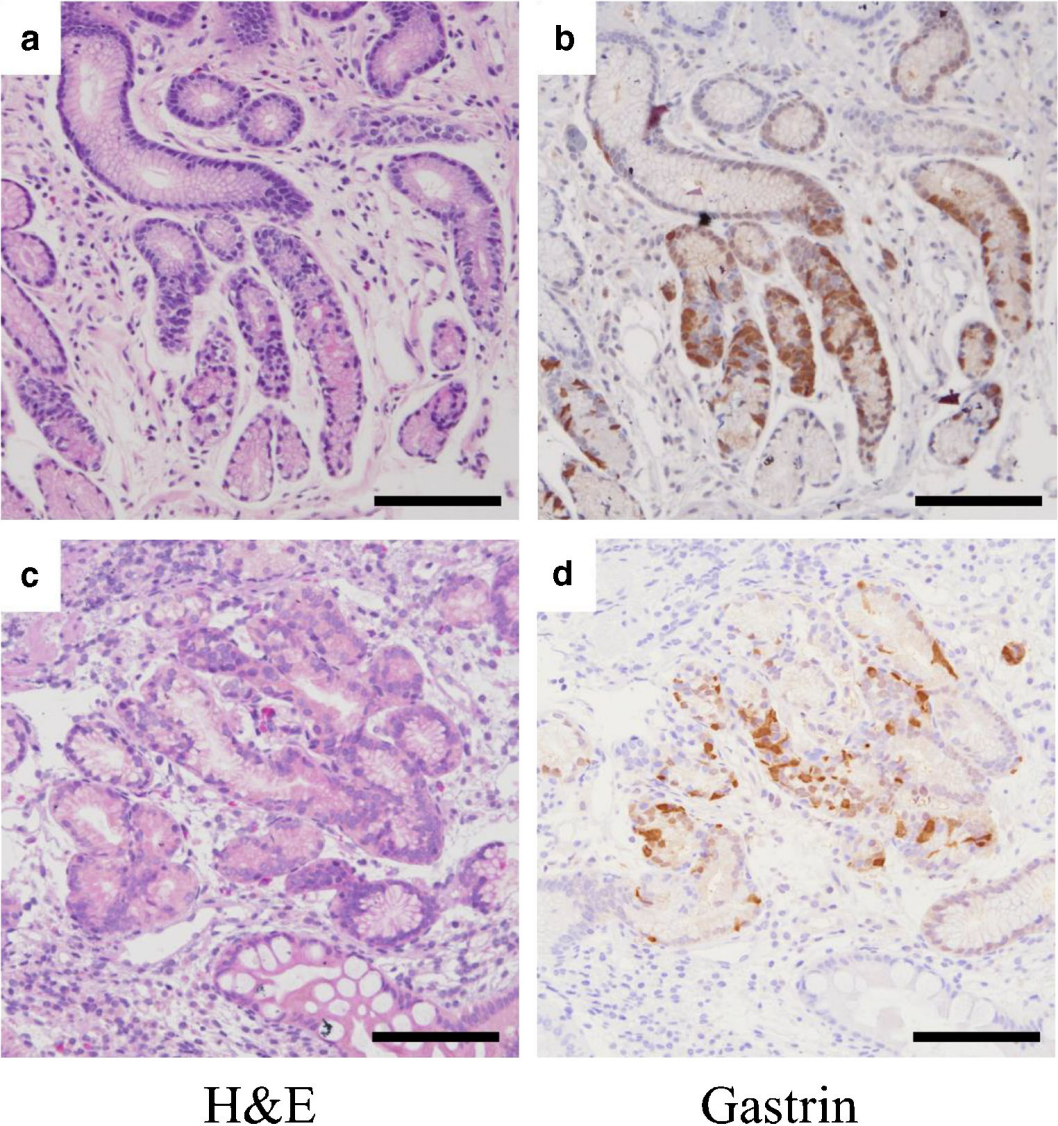
Light microscopic pictures for gastrin cells in serial sectioned slides in the antrum (**a**, **b**) and corpus (**c**, **d**) in typical AIG cases: H&E (**a**, **c**) and immunohistochemical staining with anti-gastrin (**b**, **d**). Gastrin-positive cells are recognized in the pyloric glands in the antrum (**b**). Gastrin-positive cells are also seen in the pyloric gland-like metaplasia glands in the corpus (**d**). The intensity of gastrin staining is weak in the corpus compared with that in the antrum. Scale bar: 200μm

The mean USS scores in greater curvature of the middle corpus of 22 AIG subjects were as follows: atrophy 3.0 ± 0.0, inflammation 2.1 ± 0.2, activity 0.0 ± 0.0, intestinal metaplasia 1.0 ± 0.8, and LF/LA 1.3 ± 0.7. Histopathological staging revealed that there were no cases in the early stage, 4 cases in the florid stage, and 18 cases in the end stage (Fig. 1). In greater curvature of the middle corpus, H^+^/K^+^-ATPase-positive parietal cells were found in 6 of 22 cases (4 in florid and 2 in end stages), and the mean density of the cell was significantly greater in florid stage than that in end stage (*P*<0.01, Mann-Whitney test; Table 1). Chromogranin A-positive linear and nodular ECL cell hyperplasia was observed in all cases (Fig. 2). The mean cell density was not significantly different between florid and end stages in both linear and nodular types (*P*=0.65 and 0.71 respectively, Mann-Whitney test; Table 1). BCL-10-positive pancreatic acinar cell metaplasia was not observed in florid stage, but observed in 2 cases in end stage; 1 of the cases was also stained with α-amylase but the other was not (Fig. 2). Gastrin-positive cells were found in all cases in greater curvature of the antrum, but the mean density of the cell was not significantly different between florid and end stages (*P*=0.69, Mann-Whitney test; Table 1). Gastrin-positive cells were also found in greater curvature of the middle corpus in 14 cases (1 in florid and 13 in end stage), but the intensity of gastrin staining was weak in the corpus compared with that in the antrum (Fig. 3). The mean gastrin-positive cell density in the corpus was not significantly different between florid and end stages (*P*=0.34, Mann-Whitney test; Table 1). No gastric neoplasm such as carcinoma or neuroendocrine cell tumor was found in any of the cases.

**Table 1:** Comparison of mean cell densities between stages of AIG

Immunohistochemical cell type	Mean cell densities (cells/mm^2^)		*p* value*
	Stage of AIG		
	Florid stage (*N* = 4)	End stage (*N* = 18)	
H^+^/K^+^-ATPase-positive cells in the corpus	11.18	0.33	<0.01
Linear type ECL cells in the corpus	27.63	21.63	0.65
Nodular type ECL cells in the corpus	5.45	6.40	0.71
Gastrin cells in the antrum	165.98	160.83	0.69
Gastrin cells in the corpus	3.08	5.30	0.34

The numbers of H^+^/K^+^-ATPase-positive cells, ECL cells, and gastrin cells were counted per each section. The size of each section was measured and the cell density was calculated per each section

*AIG* autoimmune gastritis, *H*^*+*^*/K*^*+*^*-ATPase* hydrogen potassium ATPase, *ECL cells* enterochromaffin cell-like cells

*Comparison between florid and end stages; Mann-Whitney test

### TFF2 expression in pyloric and pseudopyloric metaplasias

TFF2 expression in pyloric and pseudopyloric metaplasias was examined in greater curvature of the middle corpus. Pyloric metaplasia was seen in all the cases, but pseudopyloric metaplasia was found in 15 cases (Table 2). TFF2-positive pyloric gland-like glands were found in 20 cases (Table 2). Pseudopyloric metaplasia was found in all the cases in florid stage, but not all in end stage. On the other hand, TFF2-positive glands were vice vasa (Table 2).

**Table 2:** Numbers of cases containing each pyloric gland-like metaplasia in AIG

Metaplasia	Stage of AIG				total	
	Florid stage (*N* = 4)		End stage (*N* = 18)			
	No. of cases	Ratio*	No. of cases	Ratio*	No. of cases	Ratio*
Pyloric	4	100.0%	18	100.0%	22	100.0%
Pseudopyloric	4	100.0%	11	61.1%	15	68.2%
TFF2 expression	2	50.0%	18	100.0%	20	90.9%
Number of cases	4		18		22	

*AIG* autoimmune gastritis, *TFF2* trefoil family factor 2

*Ratio of cases containing each metaplasia in each stage of AIG

**Fig. 4:**
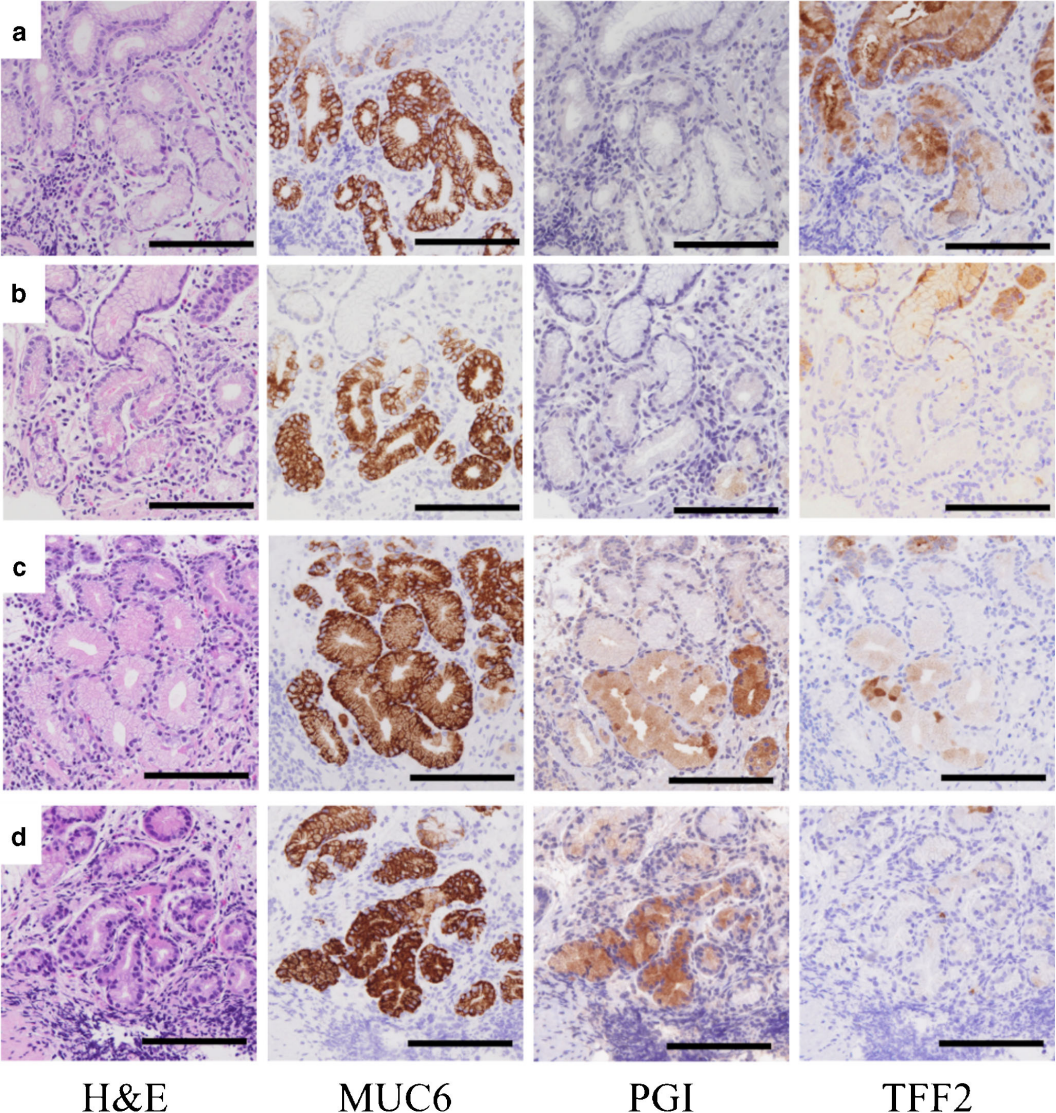
Light microscopic pictures of the four types of metaplasias in the serially sectioned slides: H&E (left pictures) and immunohistochemical stainings with anti-MUC6, PGI, and TFF2 (right 3 pictures). There were four types of glands in immunohistochemical stainings with anti-PGI and TFF2: PGI-/TFF2+ (**a**), PGI−/TFF2− (**b**), PGI+/TFF2+ (**c**), PGI+/TFF2− (**d**). Scale bar 200μm

There were 4 types of MUC6-positive metaplasia glands in the immunohistochemical stainings with PGI and TFF2 (Fig 4). Of 1567 pyloric gland–like glands in all the cases, 1381 (88.1%) glands were negative for PGI (pyloric metaplasia), and 186 (11.9%) glands were positive for PGI (pseudopyloric metaplasia). TFF2-positive and negative glands were found in both pyloric and pseudopyloric metaplasias. TFF2-positive glands were recognized in 409 of 1381 (26.9%) PGI-negative glands and 27 of 186 (14.5%) PGI-positive glands. There was a significant difference in the TFF2-positive gland ratio between PGI-negative and positive glands (*P*<0.01, chi-square test; Table 3). We also counted the numbers of pyloric gland–like glands with gastrin cells because gastrin-positive cells were found in greater curvature not only of the antrum but of corpus as mentioned above. Gastrin-positive cells in the corpus were seen in 58 of 1567 metaplasia glands. These gastrin-positive cells were only found in pyloric but not in pseudopyloric metaplasia glands. However, gastrin-positive cells were found irrespective whether the gland was TFF2-positive or not (Table 4).

**Table 3:** Total numbers of pyloric/pseudopyloric metaplasia glands with/without TFF2 expression in all the 22 cases

Metaplasia	TFF2 staining		Total	Percent/total	TFF2-positive ratio*
	+	−			
Pyloric (PGI−)	409	972	1381	88.1%	29.6%
Pseudopyloric (PGI+)	27	159	186	11.9%	14.5%
Total	436	1131	1567	100.0%	

Pseudopyloric metaplasia was idenfitied with PGI staining. The numbers of pyloric and pseudopyloric metaplasia glands were counted in all the 22 sections. Among them, the numbers of TFF2-positive and -negative glands were also counted

*TFF2* trefoil factor family 2, *PGI* pepsinogen I

*P<0.01, chi-square test

**Table 4:** Total numbers of metaplasia glands with/without gastrin staining cells in all the 22 cases

PGI staining	Metaplasia	Gastrin staining	TFF2 staining	
			+	−
−	Pyloric	+	11	47
		−	398	925
+	Pseudopyloric	+	0	0
		−	27	159

The numbers of pyloric and pseudopyloric metaplasia glands with gastrin cells were counted in all the 22 sections. Among them, the numbers of TFF2-positive and -negative glands were also counted

*PGI* pepsinogen I, *TFF2* trefoil factor family 2

## Discussion

In this study, we clearly demonstrated that TFF2-positive glands (SPEM) were not always the same as PGI-positive glands (pseudopyloric metaplasia) and were overlapped with both PGI-positive and negative glands (Table 3). In addition, TFF2-positive glands were more frequently overlapped with PGI-negative than positive glands in the AIG patients (Table 3). In other words, TFF2 expression was more frequent with pyloric metaplasia than pseudopyloric metaplasia. Although SPEM has been considered synonymous with pseudopyloric metaplasia in many papers [16–20], it is confirmed that SPEM is not the same as pseudopyloric metaplasia.

From now, the terms pyloric metaplasia, pseudopyloric metaplasia, and SPEM should be used properly. Our results strongly support the opinion that SPEM is just a term proposed from animal studies, and we should not use SPEM for human materials because TFF2 expression in pyloric and pseudopyloric metaplasias is limited [15]. Or at least, when we use the concept of SPEM in humans, SPEM should not be considered the same as pseudopyloric metaplasia.

We also showed that gastrin-positive cells were not overlapped with PGI-positive glands, and PGI-positive glands were always gastrin-negative (Table 4). TFF2-positivity was not related to gastrin-positivity. These findings suggest that gastrin cells were produced in pyloric metaplasia but not in pseudopyloric metaplasia. Because TFF2 expression has nothing to do with gastrin cells, it is also consistent with that SPEM was independent to pyloric or pseudopyloric metaplasias.

In our AIG patients, the majority of pyloric gland-like metaplasia glands were PGI-negative (88.1%, Table 3). In addition, only 61.1% cases contained pseudopyloric metaplasia in end stage in spite that all the cases contained pseudopyloric metaplasia in the florid stage. It may suggest that some pseudopyloric metaplasia glands disappeared in the progression of atrophy and that pseudopyloric metaplasia may be observed only in the limited term. Or there is a possibility that pseudopyloric metaplasia glands might have changed into pyloric metaplasia glands with the progression of atrophy. These are very similar to the findings in the metaplasias in *H. pylori* gastritis. We recently reported that atrophy was more severe in the cases with pyloric metaplasia than in those with pseudopyloric metaplasia in *H. pylori* gastritis [4]. These findings suggest that pseudopyloric metaplasia occurs first in the process of atrophy and then changed to pyloric metaplasia according to the progression of atrophy.

On the other hand, TFF2 expression was vice versa (Table 2). TFF2 expression in AIG has not been studied much [36], but investigated in *H. pylori* gastritis [17]. Xia et al. reported that TFF2 expression was found more frequently in the cases with severe atrophy. These findings support that TFF2 expression may tend to occur in the cases with severe atrophy.

We have discussed AIG and its metaplasia, though, we should mention that this study has some limitations. First, the small number of subjects was studied because AIG was still uncommon in Asians including Japanese [37, 38]. Second, the metaplastic status of gastric mucosa might be affected by past *H. pylori* infection as well as AIG because past infected patients were included in this study.

However, the following conclusion could be drawn by quantitatively and closely observing metaplastic glands in each case; SPEM is not the same as pseudopyloric metaplasia in human AIG, and the majority of metaplasia in AIG is not pseudopyloric but pyloric metaplasia.

## Supplementary Information

ESM 1 (PDF 727 kb)
