# Supplementary figures and images for: Pyloric, pseudopyloric, and spasmolytic polypeptide-expressing metaplasias in autoimmune gastritis: a case series of 22 Japanese patients
Source: Virchows Arch. 2021 Jan 30;479(1):169–78. doi: 10.1007/s00428-021-03033-5 (PMC8298345; doi:10.1007/s00428-021-03033-5)

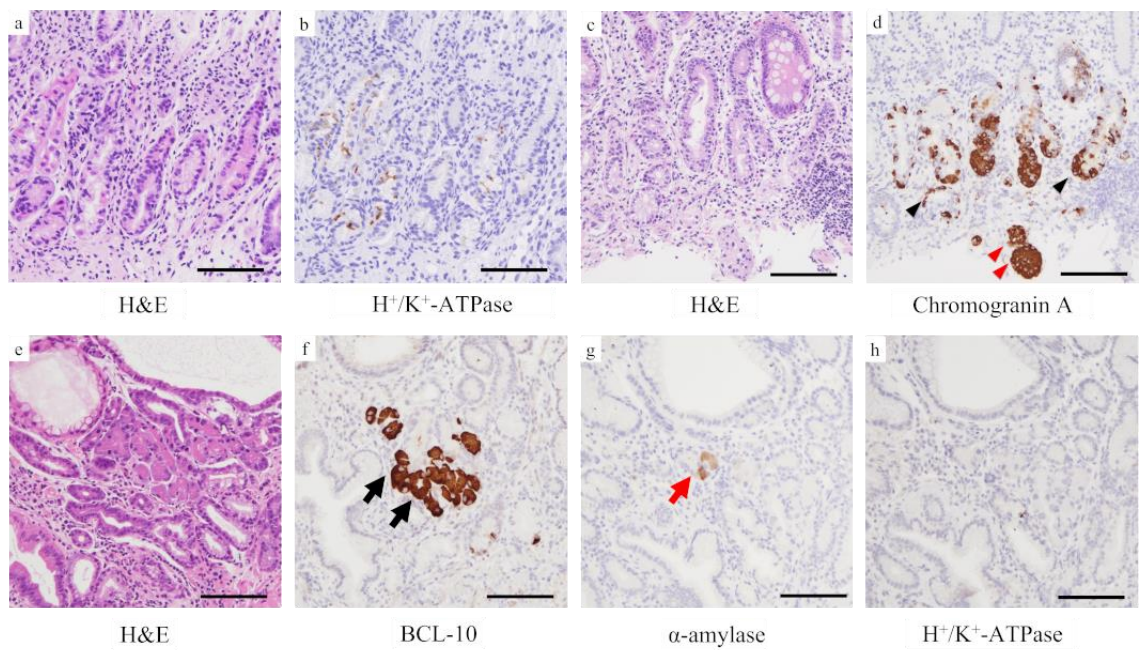

Fig.2

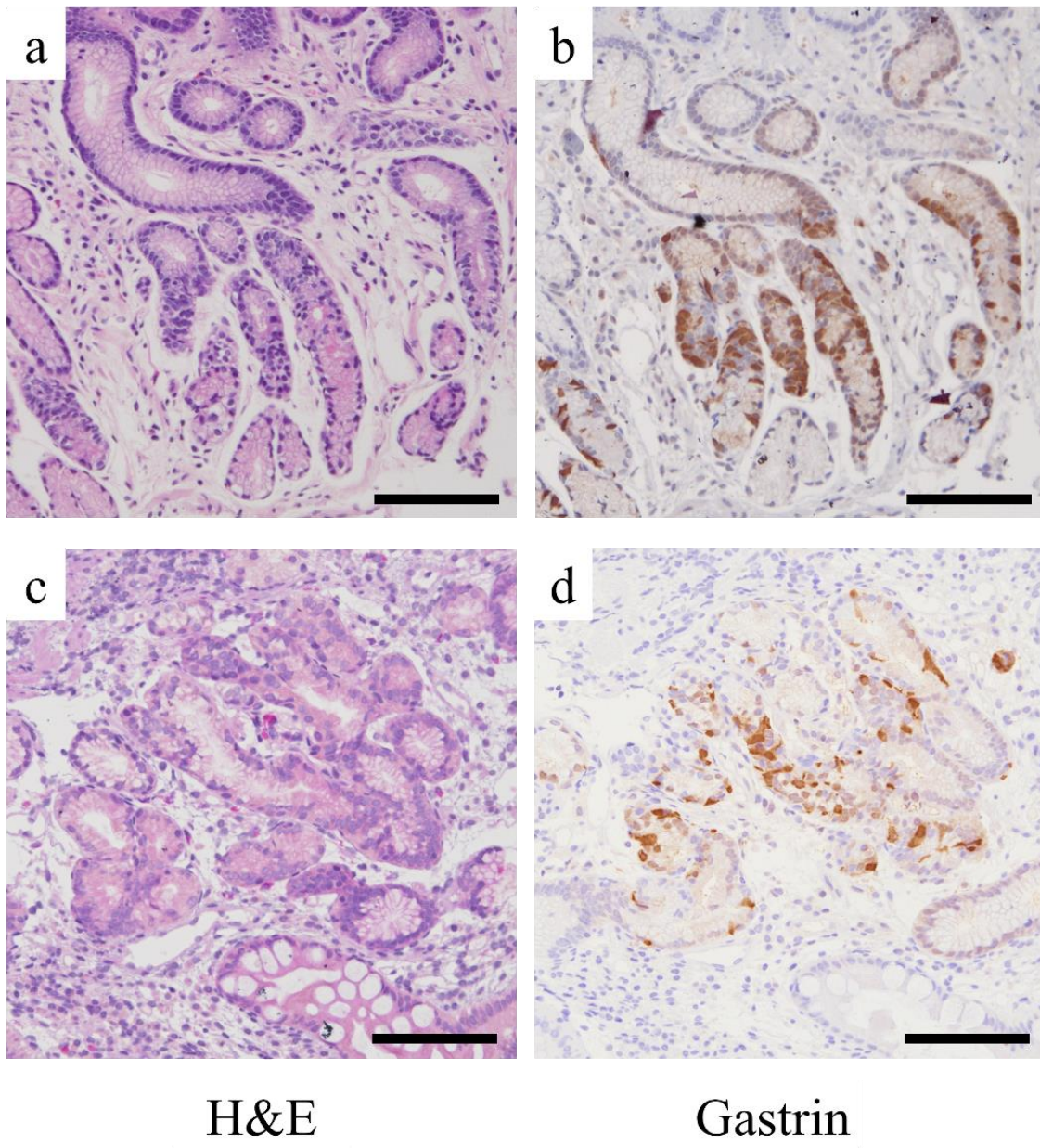

Fig. 3

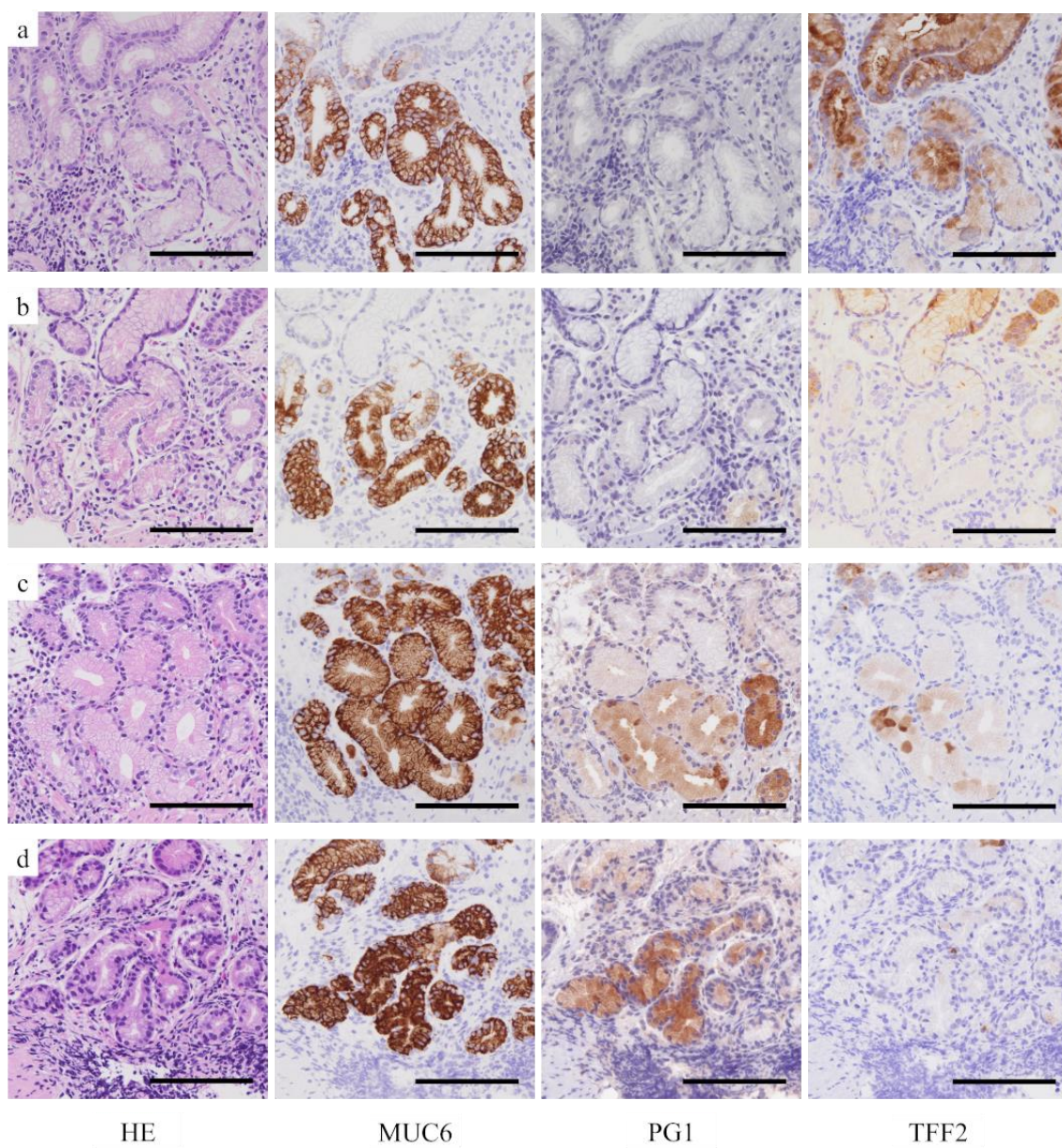

Fig. 4

Supplement: Supplementary file 1 — (PDF 727 kb) [file 428_2021_3033_MOESM1_ESM.pdf]
